# Temperature-dependent metabolic adaptation of *Triticum aestivum* seedlings to anoxia

**DOI:** 10.1038/s41598-018-24419-7

**Published:** 2018-04-18

**Authors:** Shaobai Huang, Rachel N. Shingaki-Wells, Jakob Petereit, Ralitza Alexova, A. Harvey Millar

**Affiliations:** ARC Centre of Excellence in Plant Energy Biology, Bayliss Building, M316, The University of Western Australia, 35 Stirling Highway, Crawley, WA 6009 Western Australia Australia

## Abstract

Wheat (*Triticum aestivum*) is considered anoxia intolerant but it shows variance in anoxia responses between genotypes and environmental treatments. We firstly examined 4 day old seedlings of five wheat genotypes in response to anoxia at 15 °C and 28 °C by assessing growth rate, tissue damage and changes in metabolite abundances. Significant genotypic variations in anoxia tolerance were observed, especially at 28 °C. Wheat seedlings grown at 15 °C appeared to be more anoxia tolerant and showed less genotypic variation than those at 28 °C. To minimize seedling size variations and define the temperature effects, we grew two contrasting genotypes at 15 °C for 3.5 d and adapted to 4 different temperatures for 0.5 d before exposing them to anoxia at each adapted temperature. Genotypic variation in abundance of anoxia induced metabolites occurred at 24 °C and 28 °C but not at 15 °C and 20 °C. Tissue- and temperature-dependent metabolic adaptations to anoxia were revealed. In roots, the ability to maintain sugar/sugar-phosphate and TCA cycle metabolite levels and the accumulation of amino acids when temperature was below 24 °C correlated with anoxia tolerance. Temperatures between 20 °C–24 °C are critical for metabolic adaptation and suggest that further assessment of waterlogging/flooding tolerance of wheat seedlings should consider the temperature-dependence of tolerance in evaluations.

## Introduction

Flooding, waterlogging, or high soil microbial activity cause oxygen deprivation in plants. Worldwide, floods affect 17 million km^2^ of arable land^1^. Flooding is expected to increase in frequency as a consequence of climate change and lead to yield loss for many crops^2,3^. The two major crops, rice and wheat, differ markedly in their anoxia tolerance. Rice (*Oryza sativa*) is highly tolerant to anoxia and is able to survive partly by increasing the abundance of enzymes involved in anaerobic ATP production, namely glycolysis and ethanolic fermentation^4,5^. Rice also maintains growth, even under anoxic conditions, for a sustained period of time. This tolerance in rice is underpinned by extensive alteration of primary metabolism and is also linked to tissue type as the coleoptile grows and extends under anoxia while roots stop elongation under anoxia^2,6,7^. In contrast, wheat (*Triticum aestivum*) is relatively anoxia intolerant^7–9^ and fails to rapidly adjust cytoplasmic pH and enzyme abundances in order to respond to an anaerobic environment^10,11^. For example, the increased rate of glycolysis for energy production under low oxygen is small in wheat, suggesting regulation of energy metabolism is at least partially to blame for the intolerance of wheat to O_2_ deprivation^9,12^.

Given wheat’s limited ability to withstand anoxia, the assessment of anoxia or waterlogging tolerance is distinct from studies of anoxia in tolerant species and is challenged by the lack of consistent performance of so-called tolerant varieties across variations in environmental factors such as temperature^9^. For example, field trials conducted in Mexico showed the high tolerance of the wheat variety Ducula to waterlogging; whereas trials conducted in Australia and India showed many other wheat genotypes outperforming Ducula^13^. This performance difference was suggested to be caused by different environmental conditions including temperature^13^. In another study, wheat seedlings of eleven genotypes were assessed at 15 °C for responses to anoxia and showed differences in seminal root elongation and root tissue K^+^ concentration^14^. Here, Ducula appeared to be relatively more anoxia tolerant than other wheat varieties at 15 °C^14^. In barley, varieties of Kustaa, Hankkija-673, and Pokko differed in their tolerance to anoxia and flooding at 5 °C but not at 20 °C, where all varieties displayed reduced survival^15^. Improvement of waterlogging tolerance of wheat seedlings under low temperatures has been shown to be partially due to a small relative growth rate^16^. More detailed analysis showed that wheat roots lose elongation potential at 25 °C after only 10 h of anoxia, compared to seedlings at 15 °C, where after 20 h anoxia 20–70% of the elongation potential remained^17^. It was suggested that the potential difference in ethanolic fermentation and metabolic adaption may cause this differential anoxia tolerance^17^. To date, there is no detailed information on metabolic adaption of wheat seedlings in response to anoxia at the different temperatures to explain these observations.

Plants undergo considerable metabolic changes in response to anoxia. Alanine accumulation is common in most plant species under anaerobic conditions^2,18^, and is thought to benefit pH balance within anoxic cells^19^. There are several metabolic pathways responsible for the accumulation of alanine upon anoxia. Induction of alanine amino transferase (AlaAT) activity and genes encoding AlaAT under flooding have previously been reported to contribute to alanine accumulation^20–22^. However, alanine accumulation was also observed in *AlaAT* knockout lines lacking AlaAT activity^18^. Alternatively, alanine can be produced by gamma-aminobutyric acid transaminase (GABA-T) using pyruvate as co-substrate^23^. It has also been suggested that alanine and succinate accumulation during anoxia improves ATP production during anoxia^24^.

Previously, we found that alanine failed to accumulate during anoxia in wheat coleoptiles, which was in dramatic contrast to anoxia tolerant rice coleoptiles at 28 °C^10^ and to other plant tissues as noted above. To further understand whether this alanine response was genotype-, tissue- or temperature-dependent, we firstly selected 5 wheat genotypes and 2 temperatures (15 °C and 28 °C) to measure growth, damage and metabolic response in root and coleoptile tissues. To minimize seedling size variations and define the temperature effects, we then selected two contrasting genotypes grown at 15 °C for 3.5 d and transferred them to 4 different temperatures (15, 20, 24, 28 °C) for 0.5 d and then exposed them to anoxia for 1 d. Genotype-, tissue- and temperature-dependent metabolic adaptations to anoxia were revealed. We provided evidence that changes of temperature between 20 °C–24 °C are critical for metabolic adaptation of wheat seedlings, especially root tissue, to anoxia.

## Results

### Growth under anoxia and recovery

To understand the impact of anoxia and temperature on wheat growth, we selected 5 genotypes (Calingiri, Carnamah, Spear, SARC, Ducula) and grew them at 15 °C or 28 °C continuously. We measured length of coleoptiles, leaves, primary roots and seminal roots in aerated seedlings as well as seedlings subjected to one day of anoxia followed by three days of re-oxygenation. The absolute growth data indicating tissue lengths at different time points are given in Fig. S1 with statistical analyses shown in Table S1. Overall, temperature has a significant impact on the length of all tissues measured (p-value < 0.001 for all tissues), with seedlings at 15 °C showing developmental delays when compared with seedlings at 28 °C (Fig. S1, Table S2).

**Figure 1:**
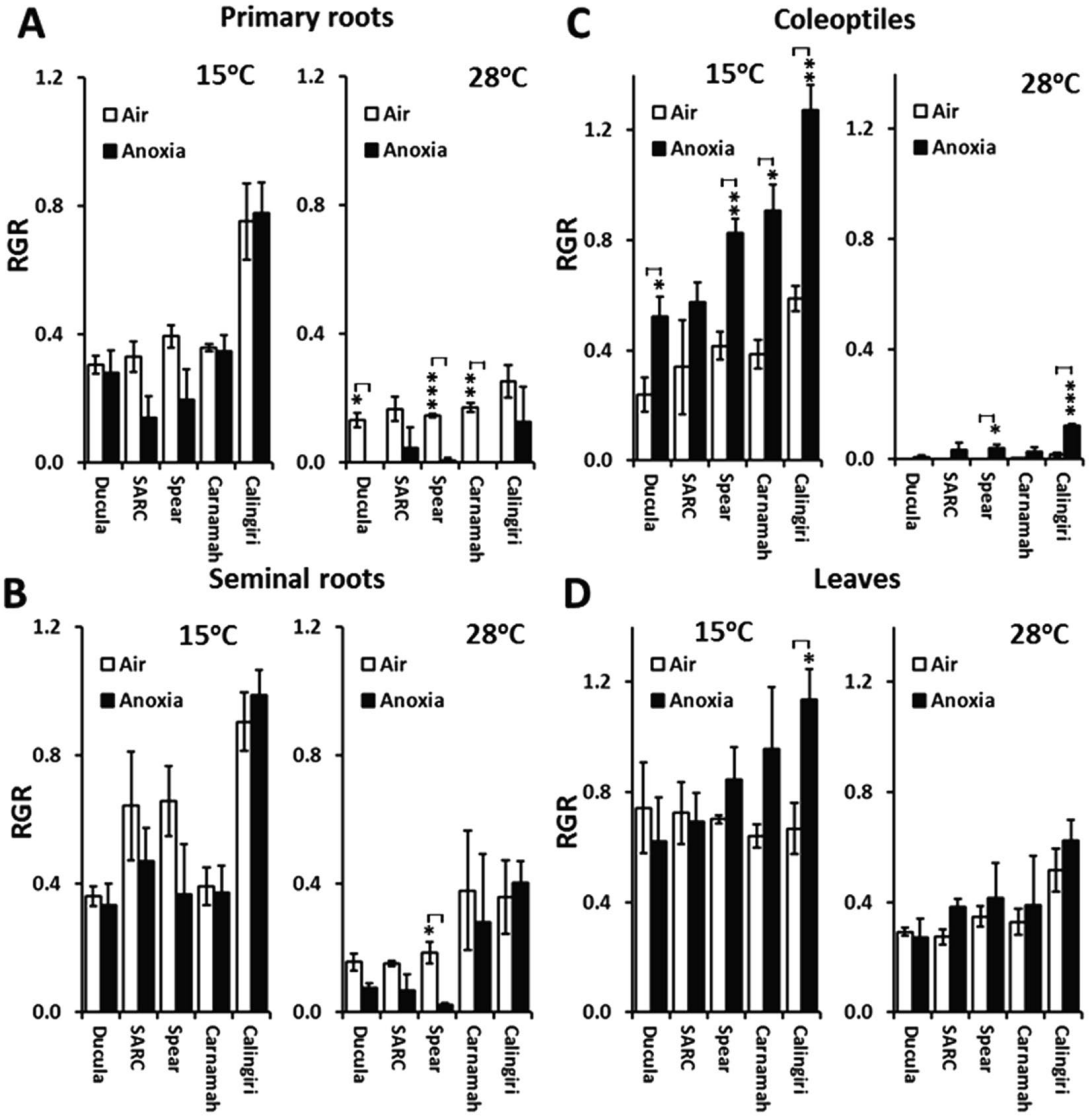
Relative growth rates of wheat tissues at 15 °C or 28 °C subjected to one day of anoxia and during recovery after they were treated with anoxia for 1 day, in comparison to aerated controls. (**A**) Primary roots (n = 10), (**B**) seminal roots (n = 10), (**C**) coleoptiles (n = 10), (**D**) Leaves (n = 10). ***Indicates p-value < 0.001; **indicates p-value < 0.01 and *indicates p-value < 0.05.

Following three days of re-oxygenation, it was revealed that the 28 °C anoxic treatment strongly inhibited the recovery of primary root elongation in Ducula, Spear and Carnamah (Fig. 1A). Only Calingiri failed to show significant anoxic growth inhibition at 28 °C. In terms of the post-anoxia recovery of seminal root elongation at 28 °C, after three days only Spear showed significant inhibition (Fig. 1B). After the 15 °C anoxic treatment, primary and seminal roots of all five cultivars showed similar growth rates as the control (Fig. 1A and B), indicating no injury during anoxia treatment at low temperature.

After the 28 °C anoxia treatment, only the coleoptiles of Calingiri and Spear showed higher proportional growth than control seedlings (Fig. 1C), while, in contrast, four of the five cultivars showed enhanced growth of the coleoptile following the 15 °C anoxia treatment. We also measured leaf elongation at a developmental stage where leaves were beginning to emerge from the coleoptiles. The anoxia treatment had less of an impact on the recovery of leaves, as indicated by the fact that proportional growth measurements after 3 days of re-oxygenation are not significantly different between anoxia treated and aerated controls (Fig. 1D), with the exception of Calingiri leaves at 15 °C which displayed a quicker growth after anoxia.

Overall, the coleoptile was the most anoxia tolerant tissue across all cultivars with Calingiri displaying the greatest response. Compared with coleoptiles, roots growth was much more severely inhibited by anoxia treatment especially in combination with a temperature of 28 °C.

### Cell injury

**Figure 2:**
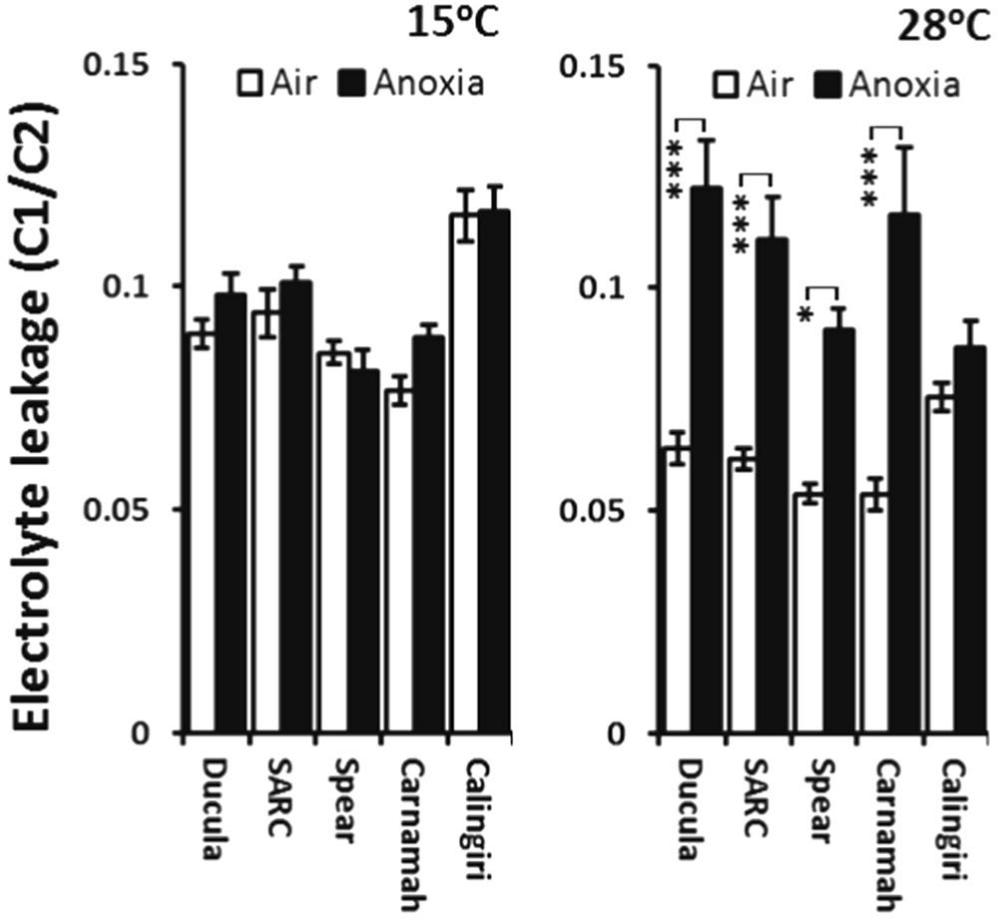
Electrolyte leakage of whole wheat seedlings at 15 °C or 28 °C subjected to one day of anoxia or control growth. ***Indicates p-value < 0.001; **indicates p-value < 0.01 and *indicates p-value < 0.05.

Growth rate alone is only a partial indicator of the damage sustained from the anoxia treatment. We measured electrolyte leakage from seedlings as an independent assay of cell damage for wheat genotypes grown at 15 °C and 28 °C (Fig. 2). The ratio of electrolyte leakage from whole seedlings after 1 h incubation in milliQ water (C1) and after boiling (C2) can be used as a proxy for cell damage^25,26^. At 28 °C, all genotypes except Calingiri had significantly higher leakage values after one day of anoxia, compared to the aerated control (Fig. 2), suggesting that Calingiri is much less damaged by anoxia than the other genotypes. At 15 °C, anoxia did not increase electrolyte leakage in any of the genotypes (Fig. 2). These results indicate anoxia cause more injury to wheat seedlings at 28 °C than at 15 °C.

### Metabolic response of coleoptiles and roots to 28 °C and 15 °C

**Figure 3:**
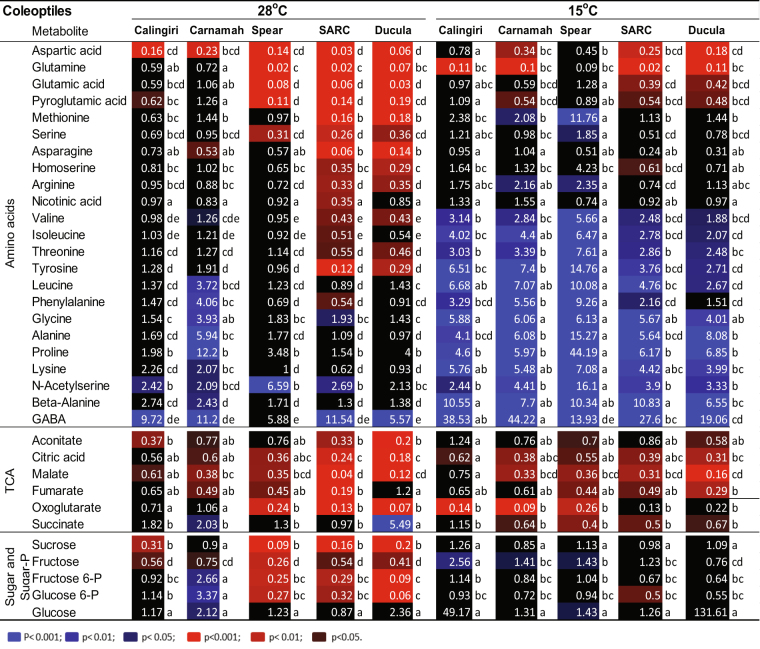
Changes of metabolites in coleoptiles of five wheat genotypes in response to 1 d anoxia at 28 °C and 15 °C. Wheat seedlings grew at 15 °C or 28 °C for 4 days and then exposed to anoxia for 1 d at 15 °C or 28 °C. The red and blue colours with different intensities indicated significant depletion and accumulation of metabolites at different P-values (p) after anoxic treatment, respectively. One-way ANOVA analysis and multiple pairwise comparisons across temperatures and genotypes (Tukey’s honest significant difference, p-value < 0.05) were conducted using XLSTAT software.

**Figure 4:**
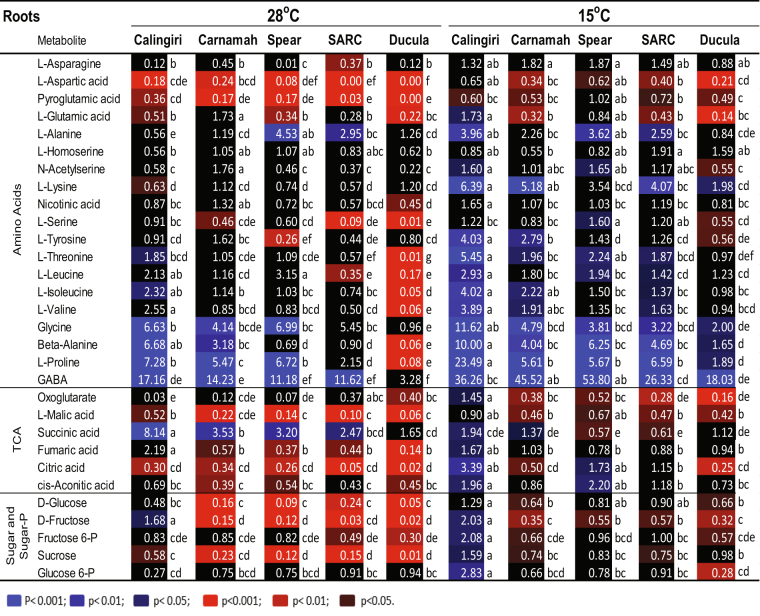
Changes of metabolites in roots of five wheat genotypes in response to 1 d anoxia at 28 °C and 15 °C. Wheat seedlings grew at 15 °C or 28 °C for 4 days and then exposed to anoxia for 1 d at 15 °C or 28 °C. The red and blue colours with different intensities indicated significant depletion and accumulation of metabolites at different P-values (p) after anoxic treatment, respectively. One-way ANOVA and multiple pairwise comparisons across temperatures and genotypes (Tukey’s honest significant difference, p-value < 0.05) were conducted using XLSTAT software.

We have previously shown an unusual non-responsiveness of alanine content in Calingiri coleoptiles to anoxia at 28 °C^10^. To explore the apparent variation in metabolite abundances in response to anoxia between genotypes and tissues, we measured metabolites in 4-day-old coleoptiles and roots at 15° and 28 °C before and after a one day anoxia treatment. We focused on three groups of metabolites, namely amino acids, TCA cycle metabolites and sugar/sugar-phosphate. The ratios of metabolite abundances after to before anoxia treatment are given in Figs 3 and 4. The ratios of change in other detected metabolites in response to anoxia are presented in Tables S3 and S4 and Supporting dataset 1 and 2. The ratios of metabolites at 15 °C vs 28 °C before anoxia treatment in coleoptiles and roots are also presented in Tables S5 and S6, respectively.

For coleoptiles at 28 °C, we observed no significant changes of alanine, nor beta-alanine, leucine, phenylalanine, glycine or proline in Calingiri, Ducula, Spear, and SARC (Fig. 3), but there was some accumulation in Carnamah. GABA was significantly accumulated after anoxic treatment across all the genotypes. After anoxia treatment at 28 °C, Ducula and SARC had much higher depletion of other amino acids than Calingiri and Carnamah (Fig. 3). A similar pattern for TCA metabolites and sugar/sugar-phosphate were also observed except for succinate and glucose (Fig. 3). Those results suggested coleoptiles of Calingiri and Carnamah at 28 °C outperformed coleoptiles of other genotypes, which is broadly consistent with the growth data (Figs 1 and S1).

For coleoptiles at 15 °C, we observed the clear accumulation of alanine alongside other major amino acids during anoxia treatment (Fig. 3). Depletions of TCA metabolites but not sugar/sugar-phosphate were also detected (Fig. 3). Overall, all genotypes showed a very similar response pattern to anoxia, indicating limited genotypic variation in metabolite levels at this temperature (Fig. 3).

For roots at 28 °C, very high accumulations of GABA, proline and glycine were observed in Calingiri, Carnamah and Spear (Fig. 4). Ducula displayed an especially high depletion of amino acid in roots, suggesting potential injury by anoxia. Most of TCA metabolites and sugar/sugar-phosphates decreased in Spear, SARC and Ducula, but remained stable in Calingiri (Fig. 4).

For roots at 15 °C, high accumulation of GABA, glycine, beta-alanine, proline and lysine was detected across 5 genotypes (Fig. 4). Other amino acids responded to anoxia in a very similar manner with a slightly higher rate of depletion in Ducula (Fig. 4). Depletion of TCA metabolites and some sugar/sugar-phosphate were also observed across genotypes with the exception of Calingiri showing accumulation pattern (Fig. 4).

It should be noted that significant differences in metabolic profile exist in tissues grown at 15 °C or 28 °C for 4 days (Tables S5 and S6), presumably caused by differences in size reached on a given day before anoxia treatment (Fig. S1, Table S2). For example, there was a significant decrease in amino acids and an increase in some TCA metabolites in coleoptiles grown at 15 °C compared with those grown at 28 °C (Table S5). In contrast, higher accumulation of amino acids and sugar/sugar-phosphates were observed in roots grown at 15 °C compared to those grown at 28 °C (Table S6), partially explaining the high anoxia-tolerance of roots at low temperature due to the availability of substrates for further energy production.

### Principle component analysis

To further explore the genotypic variations, we conducted principle component analysis using data of relative growth rate (coleoptiles, roots and leaf) and electrolyte leakage and normalised relative abundance of amino acid, TCA metabolites, sugar/sugar-phosphate before and after anoxic treatment. Assessment of these combined data sets by principal components analysis showed that 2 components could explain more than 57% of the variation in the dataset (Fig. S2). Plotting of PC1 (31%) and PC2 (26%) showed that these components clearly separated 15 °C anoxia (15 N) and 15 °C aerated (15 A) treatments, while the 28 °C treatments were much closer together. Within the 15 N and 15 A treatments it was also evident that Calingiri and Ducula were separated at the extremes of each distribution (Fig. S2).

Analysis of the top 10 positive and negative loadings for each variable in PC1 and PC2 provided a useful insight into the complex patterns with their metabolic determinants. The negative loadings in PC1 (which drew 15 N treatments away from the 28 N/28 N and 15 A treatments) were dominated by root amino acids, led by alanine and glycine abundance, and including high GABA levels (Fig. S2). In contrast the positive loadings in PC1 were mainly amino acids in coleoptiles. Positive loadings in PC2 were dominated by TCA cycle intermediate organic acids and sugars in both roots and coleoptiles (Fig. S2). Overall lower levels of organic acids at 28 °C and the lack of amino acid accumulation under anoxia underlie the difference between 28 °C and 15 °C and also the differences between two extreme varieties– Calingiri and Ducula (Fig. S2). This shows that any one metabolic abundance or anoxia response phenotype is not sufficient to explain the behaviour of a variety, but that an integrated approach is required to understand the metabolic adaptation strategies in wheat.

### Metabolic response of coleoptiles and roots at 28 °C, 24 °C, 20 °C and 15 °C

Wheat seedlings grown at 28 °C for 4 days were much larger in size than those grown at 15 °C (Fig. S1, Table S2) and showed different metabolic profiles as well (Tables S5 and S6). To minimize seedling size variations and define the temperature effects, we grew two contrasting genotypes (Calingiri and Ducula) at 15 °C for 3.5 d and then adapted seedlings to 15 °C, 20 °C, 24 °C and 28 °C for 0.5 d before anoxia treatment at these corresponding temperatures. The additional temperatures of 20 °C and 24 °C were applied to refine the critical temperature for metabolic adaptation in wheat seedlings in response to anoxia.

**Figure 5:**
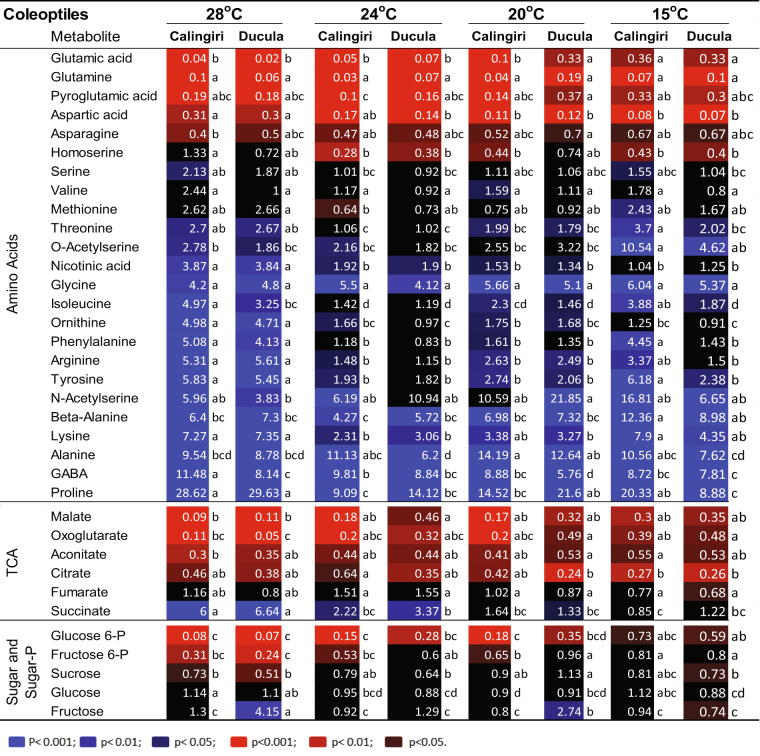
Changes of metabolites in coleoptiles of two wheat genotypes in response to 1 d anoxia at 28 °C, 24 °C, 20 °C and 15 °C. Wheat seedlings grew at 15 °C for 3.5 days and then 0.5 day at 28 °C, 24 °C, 20 °C and 15 °C before 1 day anoxic treatment at 28 °C, 24 °C, 20 °C and 15 °C. The red and blue colours with different intensities indicated significant depletion and accumulation of metabolites at different P-values (p) after anoxic treatment, respectively. One-way ANOVA analysis and multiple pairwise comparisons across temperatures and genotypes (Tukey’s honest significant difference, p-value < 0.05) were conducted using XLSTAT software.

**Figure 6:**
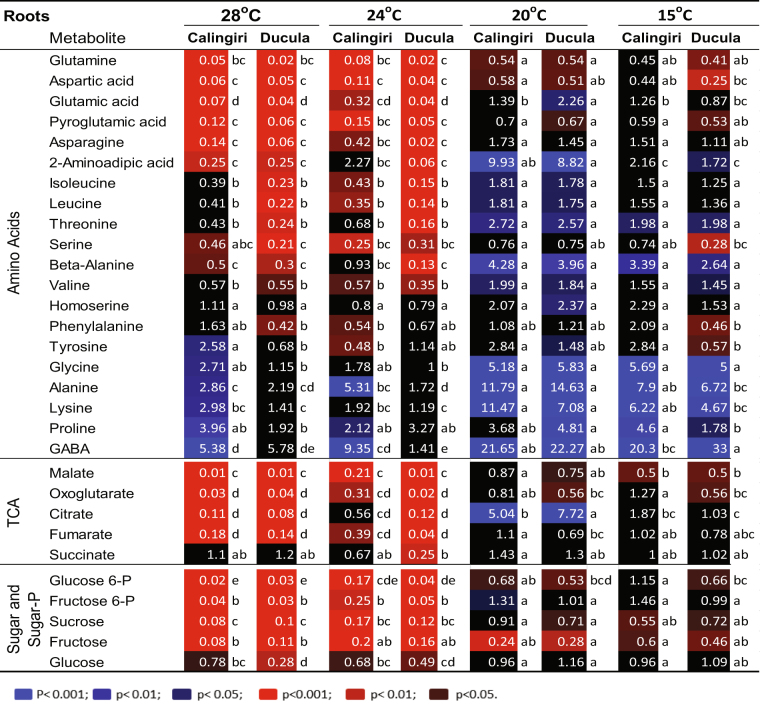
Changes of metabolites in roots of two wheat genotypes in response to 1 d anoxia at 28 °C, 24 °C, 20 °C and 15 °C. Wheat seedlings grew at 15 °C for 3.5 days and then 0.5 day at 28 °C, 24 °C, 20 °C and 15 °C before 1 day anoxic treatment at 28 °C, 24 °C, 20 °C and 15 °C. The red and blue colours with different intensities indicated significant depletion and accumulation of metabolites at different P-values (p) after anoxic treatment, respectively. One-way ANOVA analysis and multiple pairwise comparisons across temperatures and genotypes (Tukey’s honest significant difference, p-value < 0.05) were conducted using XLSTAT software.

The changes of metabolite abundance ratios in coleoptiles and roots following adaptation to the initial 12 h change in temperature to 15 °C to 20 °C, 24 °C or 28 °C were presented in the Tables S7 and S8. Overall, shifts in temperature in aerobic conditions only caused minor variations. For example, there was no variation in abundances of GABA or alanine in roots and coleoptiles of Calingiri and Ducula at different temperatures (Fig. S3). However anoxia treatment did cause large variations of tissue and temperature effects as presented in Figs 5 and 6. Other detected metabolites in coleoptiles and roots are presented in Tables S9 and S10 and Supporting dataset 3 and 4.

For coleoptiles at 28 °C, the pattern of high accumulation of amino acids in Calingiri and Ducula in response to anoxia (Fig. 5) was different from the previous pattern in relatively older coleoptiles that continuously grew at 28 °C (Fig. 3), further indicating the importance of seedling size or developmental stage for assessment of anoxia tolerance. Different temperature treatments only had minor impact on response of amino acids and TCA metabolites in both genotypes to anoxia (Fig. 5). Accumulation of succinate in coleoptiles is positively related to the increase of temperature (R^2^ = 0.80, Fig. S4). Succinate accumulation in plants in response to anoxia is widely observed^4^. Stable sugars, such as glucose and fructose, in both genotypes were observed across 4 temperatures, however sugar-phosphates such as glucose-6-P and fructose-6-P depleted with the increase of temperature (Figs 5 and S4).

In roots, however, accumulation of alanine and GABA at high temperature (24 °C and 28 °C) was observed in Calingiri but not in Ducula (Fig. 6), indicating genotypic variation between the two genotypes, which was consistent with observations when plants were grown continuously at 28 °C (Fig. 4). However, at 20 °C and 15 °C, both Calingiri and Ducula responded similarly to anoxia with high accumulation of amino acids such as GABA, alanine, lysine (Fig. 6). Accumulation of other amino acids and less depletion of glutamine and aspartic acid were also observed at 15–20 °C when compared with higher temperature treatments (Fig. 6), suggesting differential temperature response to anoxia in roots. TCA metabolites and sugar/sugar-phosphates in roots of both genotypes were dramatically depleted at higher temperatures (28 °C and 24 °C), but maintained relatively stable or only decreased slightly at low temperatures (20 °C and 15 °C) (Fig. 6), further suggesting the sensitivity of roots to adaptation of metabolites in anoxia between 20 °C and 24 °C.

### Assessment of the recovery of growth, cell injury and induction of enzymatic function during anoxia

**Figure 7:**
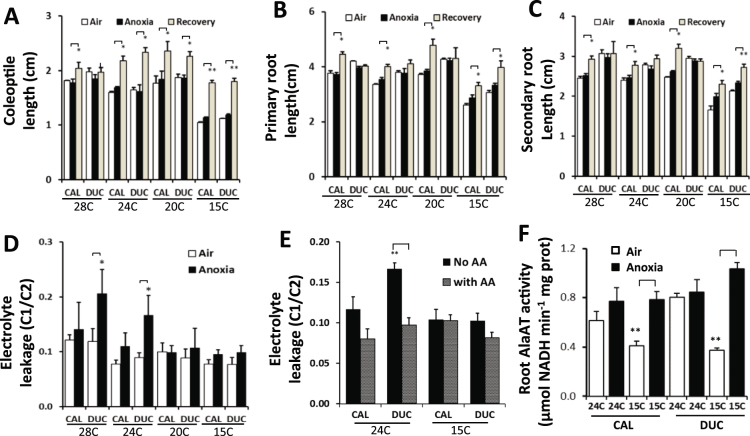
Response of wheat seedlings of two genotypes to anoxia at 28 °C, 24 °C, 20 °C and 15 °C. CAL, Calingiri; DUC, Ducula. (**A**) Coleoptile length before and after 1 d anoxia and then 1 d recovery (n = 5). (**B**) Primary root length before and after 1 d anoxia and then 1 d recovery (n = 5). (**C**) Secondary root length before and after 1 d anoxia and then 1 d recovery (n = 5). (**D**) Electrolyte leakage of wheat seedlings before and after 1 day anoxia treatment (n = 3). (**E**) Electrolyte leakage of wheat seedlings at 24 °C and 15 °C after anoxia treatment with or without AA (10 mM, alanine, glycine, serine) (n = 6). (**F**) Root alanine aminotransferase (AlaAT) activity at 24 °C and 15 °C before and after anoxia treatment (n = 4). *Indicate P value < 0.05; **indicate P value < 0.01.

To assess the damage of different tissues at different temperatures, we measured the growth of coleoptiles, primary roots and secondary roots after return to air. As shown in Fig. 7, elongation of coleoptiles, primary and secondary roots ceased to grow under anoxia at different temperatures. After return to air for 1 day, coleoptiles resumed growth with the exception of Ducula at 28 °C. For primary and secondary roots, Calingiri resumed its elongation ability during recovery with the exception of the primary root at 28 °C. In contrast, Ducula only retained elongation of roots at 15 °C during recovery.

To assess the cell injury of Ducula and Calingiri caused by anoxia at different temperatures, we measured the ion leakage from seedlings before and after anoxic treatment. Ducula but not Calingiri showed significant ion leakage at 28 °C and 24 °C after anoxia treatment, indicating cell damage in Ducula seedlings (Fig. 7D). At 20 °C and 15 °C, no obvious difference in ion leakage was found between anoxia and air treatment in both genotypes. Previously we showed that exogenous amino acids improved anoxia tolerance of wheat seedlings at 28 °C^10^. Here we selected the lower of these high temperatures, 24 °C, to test whether the addition of a combination of 10 mM alanine, glycine and serine can improve anoxia tolerance or not. The addition of this amino acid combination significantly decreased electrolyte leakage for Ducula but had no effect on the more tolerant genotype Calingiri (Fig. 7E). This indicates that endogenous accumulation or exogenous addition of amino acids under anoxia at lower ambient temperatures plays a significant role in anoxia tolerance.

To investigate a possible mechanism of endogenous changes in amino acids in roots of wheat in response to anoxia under different temperatures, we measured the activity of alanine aminotransferase (AlaAT; glutamate + pyruvate ⇌ α-ketoglutarate + alanine). Before anoxic treament, roots of both Calingiri and Ducula had higher AlaAT acitivites at 24 °C compared to those at 15 °C (Fig. 7F). At the *in vivo* level, the difference of AlaAT activity between 24 °C and 15 °C may be much greater than those at the *in vitro* detection level at 25 °C due to the Q_10_ temperature coefficient of the enzyme activity. Therefore, higher depletion of glutamate at 24–28 °C than at 15–20 °C (Fig. 6) may be the consequence of high AlaAT enzymatic activity at high temperature. Anoxia treatment did not affect AlaAT activity at 24 °C but dramatically induced its activity at 15 °C, reaching similar levels to those at 24 °C (Fig. 7F). In barley roots, two isoforms of AlaATs responded to hypoxia at 20 °C differentially^20^ indicating different functions of isoforms. Anoxia-induced increases of AlaAT activity at low temperature may be the cause of alanine accumulation that benefits for anoxia tolerance (Fig. 6).

## Discussion

Adaptation of model plants, such as rice and Arabidopsis, to anoxia has been widely studied at the transcriptomic and proteomic levels^10,27–32^. With the limitation of wheat genome annotation and the relative sensitivity of wheat to anoxia, it has been challenging to investigate adaptation of wheat seedlings to anoxia at the transcriptomic and proteomic levels. Herein, we took advantage of metabolomics analysis in short-term studies and revealed tissue-, genotype-, and most prominently, temperature-dependent metabolic adaption of wheat seedlings to anoxia.

**Figure 8:**
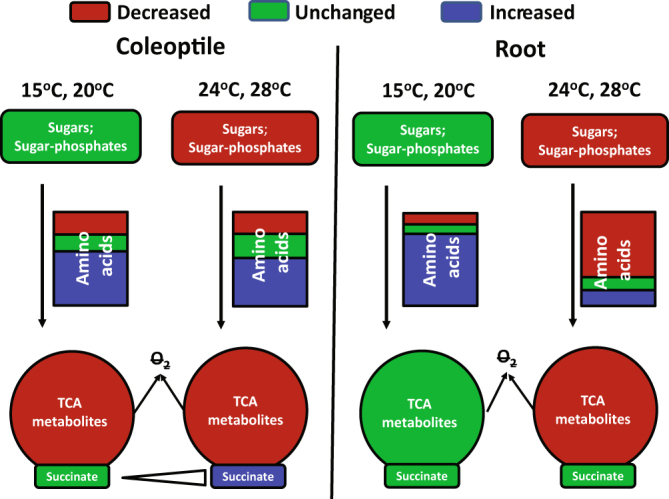
Summary of responses of wheat coleoptiles and roots to anoxia at the metabolic level under the different temperatures. Both coleoptiles and roots had depletion of sugars/sugar-phosphates at high temperature (24 °C and 28 °C) but not at low temperature (15 °C and 20 °C). Amino acids in coleoptiles respond to anoxia similarly across different temperatures. In roots, amino acids accumulated at much wider and higher level at low temperature (15 °C and 20 °C) than at high temperature (24 °C and 28 °C). In coleoptiles, depletions of TCA cycle metabolites with the exception of succinate are observed across different temperatures. In roots, depletions of TCA cycle metabolites were observed at high temperature (24 °C, 28 °C) with the exception of succinate. Overall, a change of temperature between 20 °C and 24 °C significantly shifts metabolic adaptation of wheat seedlings to anoxia, particularly in roots.

Tissue or organ specific response to low oxygen has been oberved in Arabidopsis and other plants in the context of both survival and metabolism of root and shoots^33–35^. In rice, an anoxic tolerant species, root tissues respond to anoxia by stopping elongation which is different from coleoptiles with ability of elongation under anoxia^6^. Recently more detailed analysis of metabolite profiles in *Arabidopsis* seedlings have revealed distinct response of roots and shoots^36^, with high accumulation of GABA and lactate in roots but not in shoots. Herewith, changes of amino acids in wheat coleoptiles induced by anoxic treatment were similar across different temperature treatments (Figs 5 and 8). But for roots tissues, amino acids accumulated much more broadly in lower temperatures (15 °C and 20 °C) than at higher temperatures (24 °C and 28 °C) (Figs 6 and 8). Furthermore, TCA cycle metabolites in roots were stable at lower temperatures but were depleted in coleoptile tissues (Figs 5, 6 and 8), showing the differential response of plant organs.

Assessment of anoxia tolerance in different genotypes in plants is one way to identify genetically controlled mechanisms on anoxia tolerance. Overall, Calingiri was the standout genotype in our assessment, showing tolerance to short-term anoxia with better recovery. It should be noted that re-oxygenation from anoxia commonly induced oxidative stresses^37,38^. Further research is required to test genotypic variations in the anti-oxidative system during and after anoxic treatment by measuring anti-oxidative enzymatic systems and substrates. It also appeared that genotypic variation was highly dependent on temperature. For example, at 28 °C but not at 15 °C, Ducula had more severe anoxia-induced damage as indicated by electrolyte leakage (Figs 2 and 7) and different responses to anoxia at the metabolic level in both coleoptiles and roots (Figs 3, 4, 5 and 6). In another study in which wheat seedlings were grown at 15 °C, Ducula was deduced to be more anoxia tolerant than other wheat genotypes based on measurement of K^+^ concentration and root elongation^14^. In field trails, Ducula showed high tolerance to waterlogging in Mexico but low tolerance to waterlogging in Australia and India^13^, presumably due to change of climate, including temperature. Based on our data we propose that temperature-dependent adaptation should be considered more closely for assessment of anoxia tolerance in wheat as discussed below.

Both coleoptiles and roots had depletion of sugar and sugar-phosphate at higher temperature (24 °C and 28 °C) but not at lower temperature (15 °C and 20 °C) (Fig. 8). Those results indicated the importance of sugar availability for anoxia tolerance. This is consistent with a range of literature reports. For examples, in wheat, more than 85% of roots without supply of exogenous glucose had lost their elongation potential after 9 h of anoxia compared with 30% of roots in the presence of glucose^12^. When rice coleoptiles from different genotypes with different anoxia tolerance were excised and supplied with glucose, anoxia tolerance and ethanol production rates were similar, suggesting anoxia intolerance can be a function of sugar availability^39^. In rice and pumpkin, exogenously supplied carbohydrate reduced the rate of decline in growth rate and enhanced viability of roots^40^. Two anoxia-tolerant species, *Scirpus maritimus* and *Phalaris arundinacea* consume only 0.05% of total reserve carbohydrate daily under anoxia at 20 °C, compared with 11.5% for an anoxia-intolerant species *G. maxima*, despite similar initial carbohydrate levels in the three species^41^. The slowdown of metabolism as temperature drops may contribute to the observed sugar preservation at lower temperatures in coleoptiles and roots of wheat seedlings.

Previously, we did not detect the accumulation of alanine in coleoptiles of Calingiri after anoxia treatment at 28 °C^10^. In this study, the anoxia-induced accumulation of alanine in wheat seedling was shown to be very dependent on genotype, developmental stage, temperature and tissue (Figs 3, 4, 5 and 6). Amino acids in coleoptiles responded to anoxia in a similar pattern across different temperatures (Fig. 8). In roots, amino acids, particularly GABA and alanine, accumulated at a much higher level at lower temperature (15 °C and 20 °C) than that at higher temperature (24 °C and 28 °C) (Fig. 6). Addition of exogenous amino acids also improved anoxia tolerance of wheat seedlings as indicated by less ion leakage (Fig. 7E). Accumulation of GABA and alanine in plants in response to low oxygen or anoxia has been commonly observed^42–46^. The importance of accumulation of alanine and GABA in roots and coleoptiles in all genotypes at 15 °C suggested either the operation of the GABA shunt pathway during anoxia or the blocking of the shunt leading to accumulation of GABA^47^. Depletion of glutamic acid, glutamine, pyroglutamic acid and aspartic acid can be explained by the transfer of amines to other amino acids such as alanine and GABA. Anoxia treatment dramatically induced AlaAT activity at 15 °C reaching to the similar level as those at 24 °C (Fig. 7F). In anaerobic roots of barley grown at 20 °C, the activity of alanine aminotransferase was induced over days under low oxygen stress^20^. Transamination of GABA with pyruvate produces alanine and succinic semialdehyde, which then generates succinate. When oxygen returns, alanine can be converted back to pyruvate, for eventual assimilation into aerobic metabolic processes^18,47^.

We previously observed depletion of TCA cycle metabolites, with the exception of succinate, in coleoptiles of both rice and wheat seedlings after 1 d anoxia treatment at 28 °C^10^. In this study, depletions of TCA cycle metabolites in coleoptiles, with the exception of succinate, were also observed across different temperatures and varied with genotypes (Fig. 8). The depletion of TCA cycle metabolites was presumably due to the cessation of TCA cycle operation without the respiratory electron acceptor O_2_. The positive correlation between accumulation of succinate in coleoptiles and the increase of temperature (R^2^ = 0.80; Fig. S4) indicated the temperature-dependent anoxic-induced succinate accumulation in wheat coleoptiles. It is well-known that rice shoots or coleoptiles accumulated succinate in response to anoxia^10,48,49^. In contrast to coleoptiles, TCA metabolites in roots were depleted at the two high temperatures, but remained relatively stable or only decreased moderately at two low temperatures (Figs 6 and 8). Interestingly, succinate in roots was maintained at a stable level across different temperature treatments (Figs 6 and 8). The mechanism causing these differences in metabolite abundance between coleoptiles and roots in response to anoxia awaits for further investigation.

Increase in temperature from 20 °C to 24 °C stimulated root growth of Calingiri but not Ducula, and there was no growth response for coleoptiles of either genotype (Fig. 7). For both genotypes, however, this 4 °C temperature difference (20 °C to 24 °C) caused dramatic changes in metabolic adaptation of wheat roots to anoxia (Figs 6, 7 and 8). Combinations of conservation of sugar/sugar-phosphate and TCA metabolites with accumulation of amino acids in roots when temperature was below 20 °C (Fig. 6) appeared to benefit anoxia tolerance. For example, at 25 °C, wheat roots lost elongation potential after only 10 h anoxia, but at 15 °C they maintained 20–70% of elongation potential even after 20 h anoxia^17^. During waterlogging and flooding in wheat fields, roots are the primary plant organs which suffer low oxygen or anoxia stresses. Therefore, temperature effects should be considered for the assessment of wheat waterlogging/flooding tolerance differences between genotypes.

In conclusion, we assessed 4 days old seedlings of five wheat cultivars and revealed that wheat seedlings grown at 15 °C were more anoxia tolerant than those at 28 °C. Calingiri and Carnamah outperformed SARC and Ducula for anoxia tolerance, especially at 28 °C. By adding two more temperature treatments, variations in anoxic induced metabolite changes in response to anoxia between Calingiri and Ducula could be pinpointed to occur at 24 °C and 28 °C but not at 15 °C or 20 °C. Tissue-dependent and temperature-dependent metabolic adaptations to wheat anoxia were thus revealed (Fig. 8). Conservations of sugar/sugar-phosphate, TCA metabolites and accumulation of amino acids in roots when temperature is below 24 °C contributed to the improved anoxia tolerance. We provided evidence that a temperature shift between 20 °C and 24 °C is thus critical for induced metabolic adaptation of wheat seedlings to anoxia, especially in root tissues. Consideration of temperature effects is therefore suggested for designing the assessment of wheat waterlogging/flooding tolerance.

## Methods

### Plant Material

Ducula, SARC, Spear, Carnamah have been previously tested in response to anoxia^14^. In field trails, Ducula had shown high tolerance to waterlogging in Mexico but low tolerance to waterlogging in Australia and India^13^. Calingiri was used in our previous study to compare with anoxia-tolerant species rice^10^. Wheat genotypes of Ducula, SARC, Spear, Carnamah, Calingiri were kindly provided by the Department of Agriculture and Food of Western Australia (DAFWA).

### Plant growth condition and treatments

Plants were grown according to our previous study^10^. Briefly, approximately fifty seeds of 5 genotypes (Ducula, SARC, Spear, Carnamah, Calingiri) were placed in a 250 mL growth vessel. Seeds were sterilised by adding 6% [w/v] NaOCl for 10 min. After rinsing three times with ddH_2_O, 200 ml culture solution was added (0.5 mM 2-(N-morpholino) ethanesulfonic acid, 0.4 mM CaSO_4_, pH 6.5). Lids containing a gas delivery tube and a gas outlet hole were screwed on tightly. Humidified compressed air was bubbled throughout each vessel for 4 days, in dark growth chamber with temperature of 28 °C or 15 °C. Anoxic treatments lasted for one day, by using humidified high purity nitrogen gas. The ethanol concentrations measured using enzymatic method^39^ after 1 day of anoxia treatment at 28 °C and 15 °C were 2.5 ± 0.3 mM and 0.8 ± 0.2 mM, respectively. Post-anoxic treatments were obtained by bubbling with humidified compressed air for 1 or 3 days. For another experiment with 4 temperature treatments (28 °C, 24 °C, 20 °C and 15 °C), two genotypes (Ducula and Calingiri) with contrasting response were grow in aeration for 3.5 d at 15 °C and were transferred to 28 °C, 24 °C, 20 °C and 15 °C for 0.5 d of adaptation before anoxic treatment. The seedlings were treated for 1 d of anoxia and then 1 d of recovery in aeration at the corresponding 4 temperatures.

### Growth of seedlings before and after anoxia and recovery

Root (primary root that emerged firstly after germination and seminal roots), leaf and coleoptile lengths were measured using a ruler at several stages of development with or without an anoxic treatment. There were 5–10 seedlings for each genotype/temperature/treatment combination; and each experiment was repeated three times. For the experiment of 15 °C and 28 °C treatment with 5 genotypes (Fig. S1), we measured initial lengths after 4 days in aeration and then 1 d of anoxia (Anx5d). Growth recovery from 1 d and 3 d of re-aeration (Re-ox6d and Re-ox8d) were also measured. As an aerated control, we measured seedling growth after 5 d (Air5d), 6 d (Air 6d) and 8 d (Air 8d) of aeration. The 3 days of relative growth rate after anoxia treatment was calculated as: [(Re-ox8d) - (Anx5d)]/(Anx5d) *100. The 3 days of RGR of aerated control was calculated as: [(Air8d) - (Air5d)]/(Air5d) *100. For another experiment with two contrast genotypes (Ducula and Calingiri), we measured initial lengths of coleoptiles and roots after being aerated for 3.5 d at 15 °C and transferred to 28 °C, 24 °C, 20 °C and 15 °C for 0.5 d. We also measured lengths of coleoptiles and roots after 1 d anoxia treatment and 1 d recovery to aeration at the corresponding four temperatures.

### Metabolite extraction and GC-MS analysis

Metabolites from roots and coleoptiles (without primary leaf inside) before and after anoxia treatment were extracted by placing 25 ± 5 mg tissue into 2 mL Eppendorf tubes containing a stainless steel grinding bead. Samples were snap frozen in liquid nitrogen. Tubes were placed in a liquid nitrogen-cooled mill rack for homogenisation twice. Cold 0.5 mL metabolite extraction medium (85% methanol, 15% H_2_O, and 100 µg mL^−1^ ribitol) was added to each sample and then mixed at 1400 rpm for 20 min at 65 °C using a thermomixer. Samples were centrifuged at 20,000 × g for 3 minutes at room temperature. 60 µL of supernatant was transferred to a low-volume insert and this was dried down in a vacuum centrifuge. Inserts were transferred to 2 mL eppendorf tubes for storage at −80 °C. For derivatisation, 20 µL of methoxyamine hydrochloride in anhydrous pyridine (20 mg mL^−1^) was added to each sample for incubation at 30 °C for 90 min at 1400 rpm. Then, 30 µL MSTFA was added and incubated at 37 °C for 30 min at 1400 rpm. After this, 10 µL of an n-alkane mix at 0.029% (n-dodecane, n-pentadecane, n-nonadecane, n-docosane, n-octacosane, n-dotriacontane, and n-hexatriacontane) was added. Samples were incubated at room temperature for 30 minutes prior to GC-MS analysis, the methods of which have been described previously^50^. Briefly, 1 µL of sample was injected into an Agilent 7890 GC fitted with an Agilent 5975 MSD. The carrier gas, helium, had a constant flow of 1 mL per minute. The inlet temperature was 300 °C and the initial oven temperature was set at 70 °C for 1 min. The oven temperature was increased to 76 °C at 1 °C per minute, then to 325 °C at a rate of 6 °C per minute. This temperature was held for 8 minutes. The capillary column used was a Varian Factor 4 (VF-5ms, 30 m x 0.25 mm, 0.25 µm; 10 m EZ-Guard). The MSD transfer line heater was set at 300 °C, the MS quadrupole at 150 °C and the source at 230 °C. The mass detection range was set at 40–600 atomic mass units. The generated data was collected using Chemstation GC/MSD Data Analysis Software (Agilent Technologies). Raw GC-MS data preprocessing and statistical analysis were performed using MetabolomeExpress software (version 1.0; http://www.metabolome-express.org). Detailed methods have been reported^51^.

### Cell leakage assays

Cell leakage assays were adapted from a previous study^26^. Three seedlings were submerged in 25 mL H_2_O in a 50 mL falcon tube. Samples were placed in the dark at room temperature (approximately 19 °C) for 1 hour. Samples were gently mixed and electrical conductivities measured and recorded as C1. Samples were boiled for 2 mins, then placed in an icebox for 20 minutes and allowed to equilibrate to room temperature. A second measurement of electrical conductivity was taken (C2). The ratio of C1 to C2 was calculated as a proxy for cell leakage. For amino acid treatment, mixtures of 10 mM alanine, glycine and serine were added to the culture solution before anoxic treatment.

### Alanine Aminotransferase activity assay

Root tissues were harvested before and after anoxia at 24 °C or 15 °C. Roots were ground with a mortar and pestle in five volumes of 50 mM Tris/HCl (pH 7.5) containing 1 mM DTT at 4 °C. The homogenate was centrifuged at 10,000 g for 20 min at 4 °C, The supernanants were collected and used for the alanine aminotransferase activity assay as previously described^21^. The reaction solution in 3 mL contained 10 mM L-alanine, 5 mM oxoglutarate, 5 units of lactate dehydrogenase type v-s from rabbit muscle, 0.1 mM NADH and 50 mM Tris-HCl (pH 8.0). After adding extract, the absorbance at 340 nm was recorded over 120 s using a spectrophotometer at a temperature of 25 °C. Total protein content of the enzyme extract was measured using Bradford reagent^52^.

### Principle component analysis

Principle component analysis was performed using MEV v 4.9.0, centred on medians, with the k-nearest neighbour imputation set at the default value of 10. Samples were clustered in the analysis and measurement loadings in eigenvectors were extracted for analysis and presentation. Data of relative growth rate (coleoptiles, roots and leaf) and electrolyte leakage and normalised relative abundance of amino acid, TCA metabolites, sugar/sugar-phosphate before and after anoxic treatment (see details in Supporting Dataset) were used for analysis.

## Electronic supplementary material

Supplemental Figures

Supplemental Tables1-10

Supporting Dataset1-4
