# Supplementary material for: Temperature-dependent metabolic adaptation of Triticum aestivum seedlings to anoxia
Source: Sci Rep. 2018 Apr 18;8:6151. doi: 10.1038/s41598-018-24419-7 (PMC5906562; doi:10.1038/s41598-018-24419-7)
Supplement: Supplementary file 1 — Supplemental Figures [file 41598_2018_24419_MOESM1_ESM.pdf]

Supplementary Figures for "Temperature-dependent metabolic adaptation of *Triticum aestivum* seedlings to anoxia" **(**Shaobai Huang[1] *, Rachel N. Shingaki-Wells [1], Jakob Petereit [1], Ralitza Alexova [1], & A. Harvey Millar [1])

Supplementary Figures:

Supplementary Figure 1. Tissue length of wheat seedlings at 15°C or 28°C subjected to one day of anoxia and three days of re-oxygenation, relative to continuously aerated controls. These experiments were performed three times, allowing the calculation of relative growth rate in Figure 1 (n=10). Detailed statistics analysis of one-way ANOVA and multiple pairwise comparisons (Tukey's honest significant difference , p-value< 0.05, using XLSTAT software) of different tissues across varieties were presented in Supplementary Table 1.

Supplementary Figure 2. Principal components analysis plot of PC1 vs PC2 for metabolite, growth rates, electrolyte leakage of wheat seedlings grown in air (green) or treated with anoxia (red) at 15° C or 28° C. The positive and negative loadings of variables that contribute the most to PC1 and PC2 are shown in the tables, colours of loadings indicate range of values in the top 10 loadings from highest (green) to lowest (red) impact. CL: Calingiri; CN: Carnamah; SP: Spear; SA: SARC; DU: Ducula.

Supplementary Figure 3. Normalised abundances of alanine, GABA, glucose-6-phosphate and malate in roots or coleoptiles of Calingiri and Ducula before and after 1 day of anoxia treatment (Anoxia) at 15°C, 20°C, 24°C and 28°C (n=5). The metabolite abundances have been normalised with the mean of metabolite abundances before and after anoxia treatment. The ratios of alanine, GABA, glucose-6-phosphate and malate post- anoxic treatment to pre-anoxia treatment were presented in Figures 5 and 6. One-way ANOVA and multiple pairwise comparisons (Tukey's honest significant difference , p-value< 0.05) were conducted using XLSTAT software.

Supplementary Figure 4. Correlation between anoxic-induced ratios of selected metabolites in wheat coleoptiles and temperatures from 15°C to 28°C**.** Metabolites are glutamic acid, succinate, oxoglutarate, fructose-6-P and glucose-6-P. Data are extracted from Figure 5.

Supplementary Tables1-10 in excel file are presented in supporting information:

Supplementary Table 1: Statistics analyses of all pairwise comparisons of tissue length across five genotypes at the different time points as shown in Fig S1.

Supplementary Table 2: Statatistic analyses of temperature effects on length of different tissues of 5 wheat genotypes grown at 28°C or 15°C for 4 days**.**

Supplementary Table 3: Changes of metabolite ratios in coleoptiles of five wheat genotypes in response to 1 d anoxia at 28°C and 15°C**.**

Supplementary Table 4: Changes of metabolite ratios in roots of five wheat genotypes in response to 1 d anoxia at 28°C and 15°C**.**

Supplementary Table 5: Changes of metabolite ratios in coleoptiles of 5 wheat genotypes continously grown in 15°C and 28°C for 4 days**.**

Supplementary Table 6: Changes of metabolite ratios in roots of 5 wheat genotypes continously grown in 15°C and 28°C for 4 days**.**

Supplementary Table 7: Changes of metabolite ratios in coleoptiles of two wheat genotypes in adaptation to 0.5 d of 28°C, 24°C, 20°C and 15°C.

Supplementary Table 8: Changes of metabolite ratios in roots of two wheat genotypes in adaptation to 0.5 d of 28°C, 24°C, 20°C and 15°C.

Supplementary Table 9: Changes of metabolite ratios in coleoptiles of two wheat genotypes in response to 1 d anoxia at 28°C, 24°C, 20°C and 15°C.

Supplementary Table 10: Changes of metabolite ratios in roots of two wheat genotypes in response to 1 d anoxia at 28°C, 24°C, 20°C and 15°C.

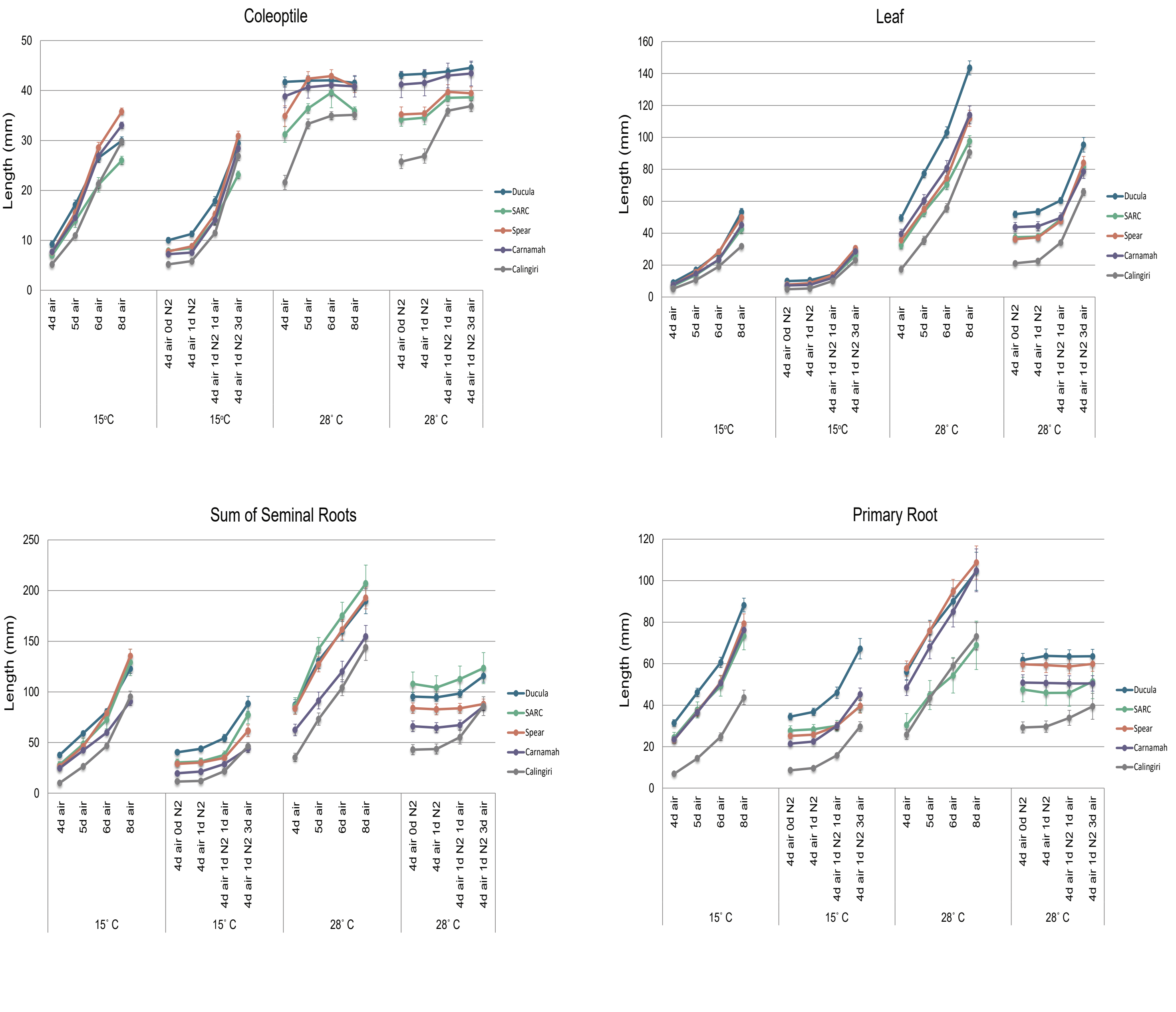

Supplementary Figure 1

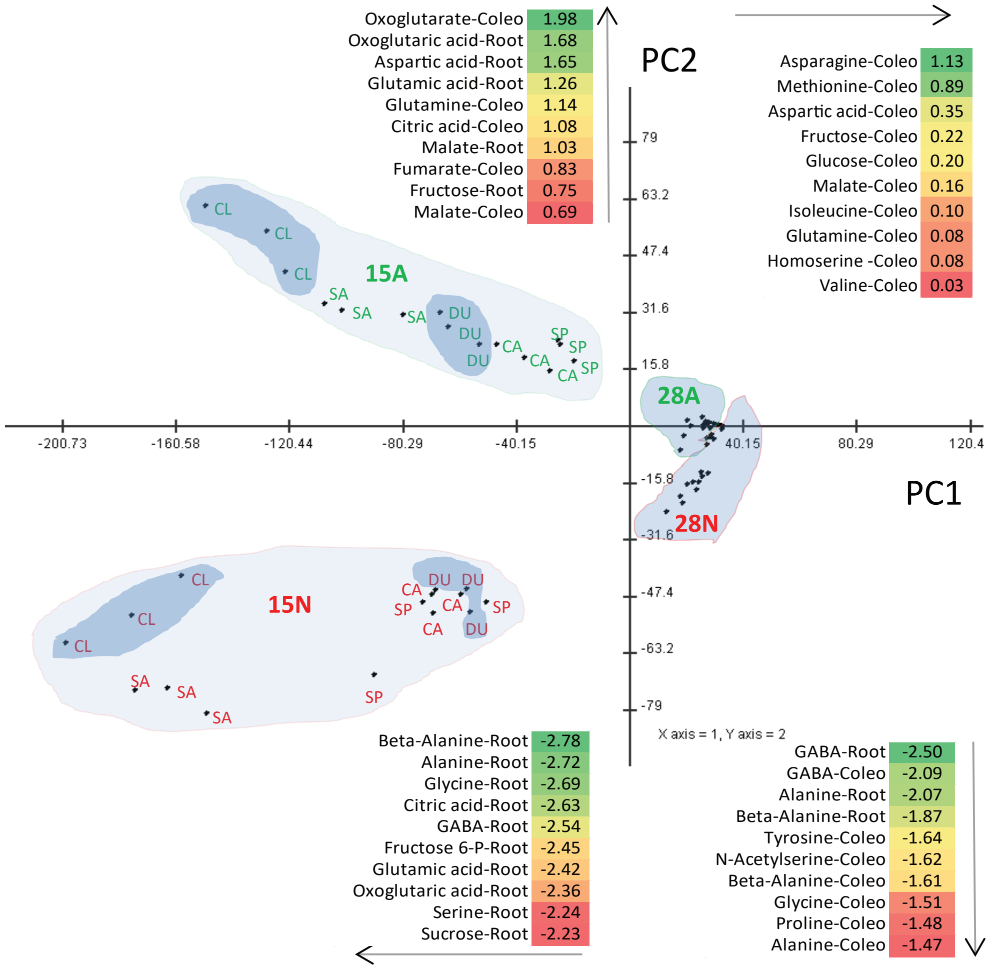

Supplementary Figure 2

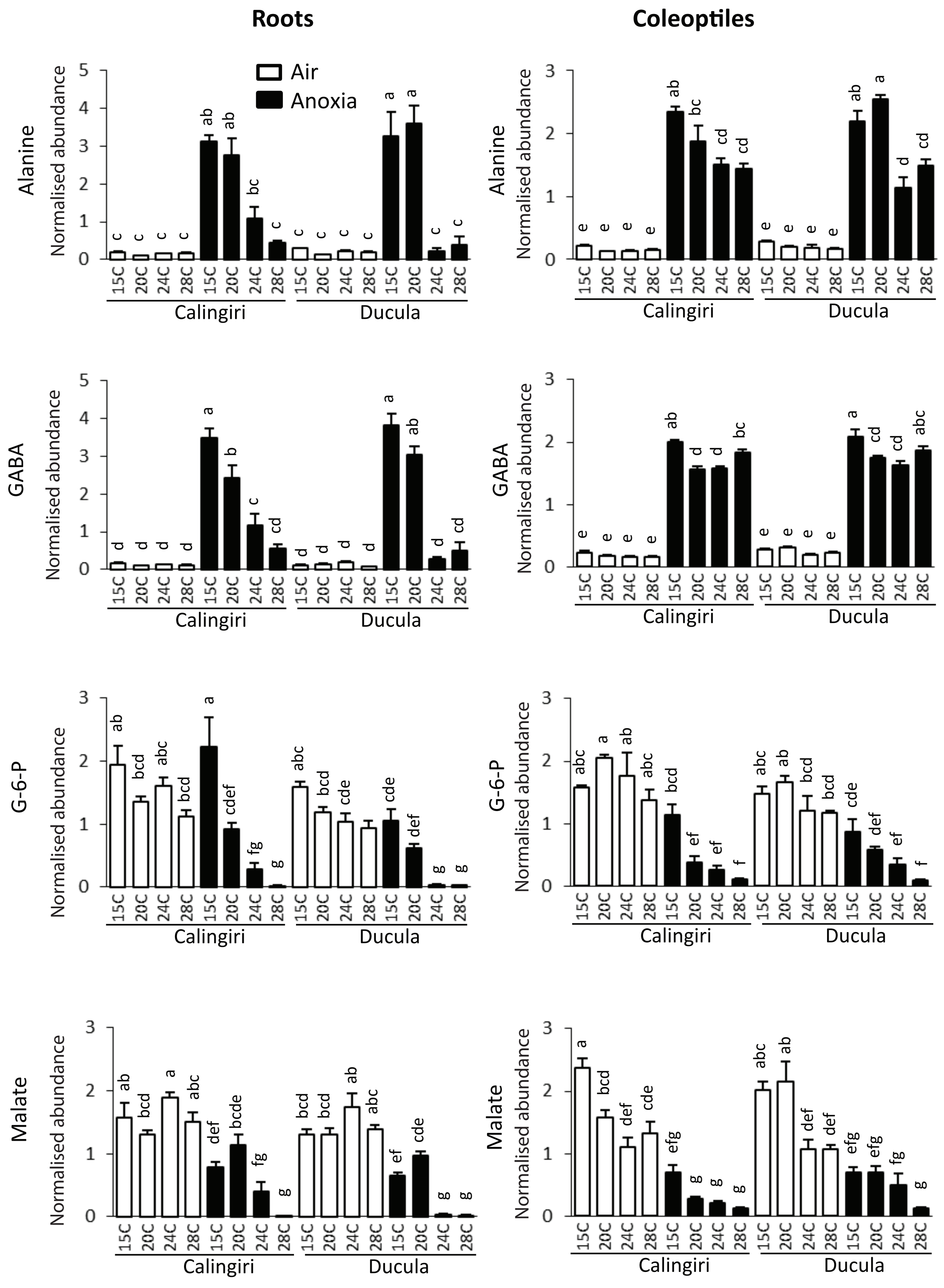

Supplementary Figure 3

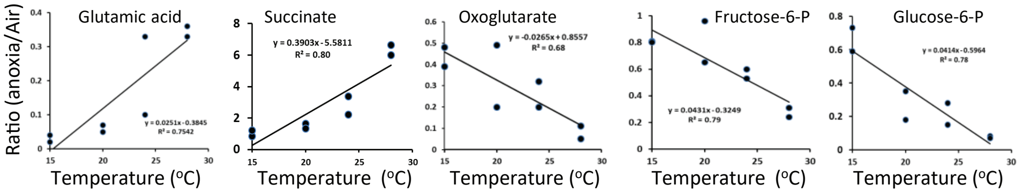

Supplementary Figure 4
